# Clinical perspectives and therapeutic strategies: pediatric autoinflammatory disease—a multi-faceted approach to fever of unknown origin of childhood

**DOI:** 10.1186/s41232-022-00204-y

**Published:** 2022-07-02

**Authors:** Akihiro Yachie

**Affiliations:** Division of Medical Safety, Kanazawa University Hospital, 13-1 Takaramachi, Kanazawa, 920-8641 Japan

**Keywords:** Autoinflammatory disease, Familial Mediterranean fever, Systemic juvenile idiopathic arthritis, Cytokine profiling, Diagnostic odyssey

## Abstract

Among the different etiologies for fever of unknown origin in children, infectious diseases are the most frequent final diagnosis, followed by autoimmune diseases and malignancies. Autoinflammatory diseases are relatively rare among children and are frequently overlooked as differential diagnoses for fever of unknown origin. Once the possibility of a particular autoimmune disease is considered by physicians, the diagnosis might be easily made by a genetic approach because many of autoinflammatory diseases are of monogenic origin. To reach the diagnosis, detailed history-taking, precise physical examinations, and cytokine profiling as well as extensive mutation analysis of candidate genes should be undertaken for febrile children. Such the approach will protect the patients, and their family to undergo “diagnostic odyssey” in which unnecessary and sometimes risky diagnostic and therapeutic interventions are taken.

This short review discusses the clinical and laboratory features of familial Mediterranean fever and systemic juvenile idiopathic arthritis, as representative illnesses of monogenic and polygenic autoinflammatory diseases, respectively. Cytokine profiling and mutation analyses both help to understand and decipher the heterogeneous pathologies in both disease categories.

## Background

Fever is one of the most common manifestations of childhood illness. In most cases, fever is a sign of acute infectious disease and subsides spontaneously without the need for specific intervention [1]. However, not infrequently, febrile episodes are prolonged or recur and the child suffers from a long-term morbidity. Although most of these febrile illnesses are due to acute or chronic infections, other disease categories should be considered in some cases as the primary cause of fever.

In addition to infectious diseases, malignancies and autoimmune diseases are the two other major categories of illness responsible for fever of unknown origin (FUO). Various studies have described the etiologies of FUO under different situations. In addition to malignancies and autoimmune diseases, the novel category of febrile disease was proposed more than 20 years ago by McDermott et al. [2]. Autoinflammatory diseases were initially described as a family of disorders characterized by a genetic predisposition to excessive inflammation, mainly reflecting overactivation of the innate immune responses, without direct involvement of the acquired immune system, such as high titers of autoantibodies or the presence of antigen-specific T cells. More recently, autoinflammatory diseases have been defined as “clinical disorders marked by abnormally increased inflammation, mediated predominantly by cells and molecules of the innate immune system, with a significant host predisposition” [3].

In this short review, the author describes the two autoinflammatory diseases most prevalent among children, namely, familial Mediterranean fever (FMF) and systemic juvenile idiopathic arthritis (sJIA), and discusses the role of physicians in employing a multi-faceted approach to reach an early diagnosis and timely application of the appropriate therapeutic interventions to avoid an unnecessary diagnostic odyssey. FMF is a prototype monogenic autoinflammatory disease, typically with a very young onset. Furthermore, FMF is highly prevalent among the autoinflammatory diseases and many patients are suggested to remain undiagnosed and untreated [4]. As a polygenic autoinflammatory disease, sJIA is particularly prevalent among Japanese children as compared to other countries, under the umbrella diagnostic term of JIA. An appropriate understanding of the pathophysiology of this potentially life-threatening illness is of paramount importance to pediatricians treating children presenting with febrile illnesses.

## Main text

### Diagnostic odyssey

Significant numbers of children have to go through a long and potentially agonizing experience during which no diagnosis is reached, or at best, an incorrect diagnosis is made. Not only is the diagnosis mistaken, but due to this misdiagnosis, unnecessary or even harmful procedures and inappropriate interventions might be performed [5]. With the development of sophisticated diagnostic techniques, including extensive mutation analysis using whole-genome sequencing and precise evaluation of the pathology, increasing numbers of novel diseases have been defined in recent years. For example, although only a handful of diseases were known within the category of primary immunodeficiency diseases 40 years ago, more than 400 diseases are now recognized within this same category [6]. Notably, many of the monogenic diseases defined as primary immunodeficiency diseases are at the same time included within the category of monogenic autoinflammatory diseases [7]. Among novel diseases and disease categories, those categorized as autoinflammatory diseases are major illnesses suffered by patients with “syndrome without a name” [8]. Such children may visit multiple clinics and hospitals until the correct diagnosis is finally reached. In many cases, decades may pass before the correct solution is obtained. Such a “diagnostic odyssey” is particularly common among children with rare diseases [9].

### Prolonged or recurrent fever without diagnosis

**Fig. 1:**
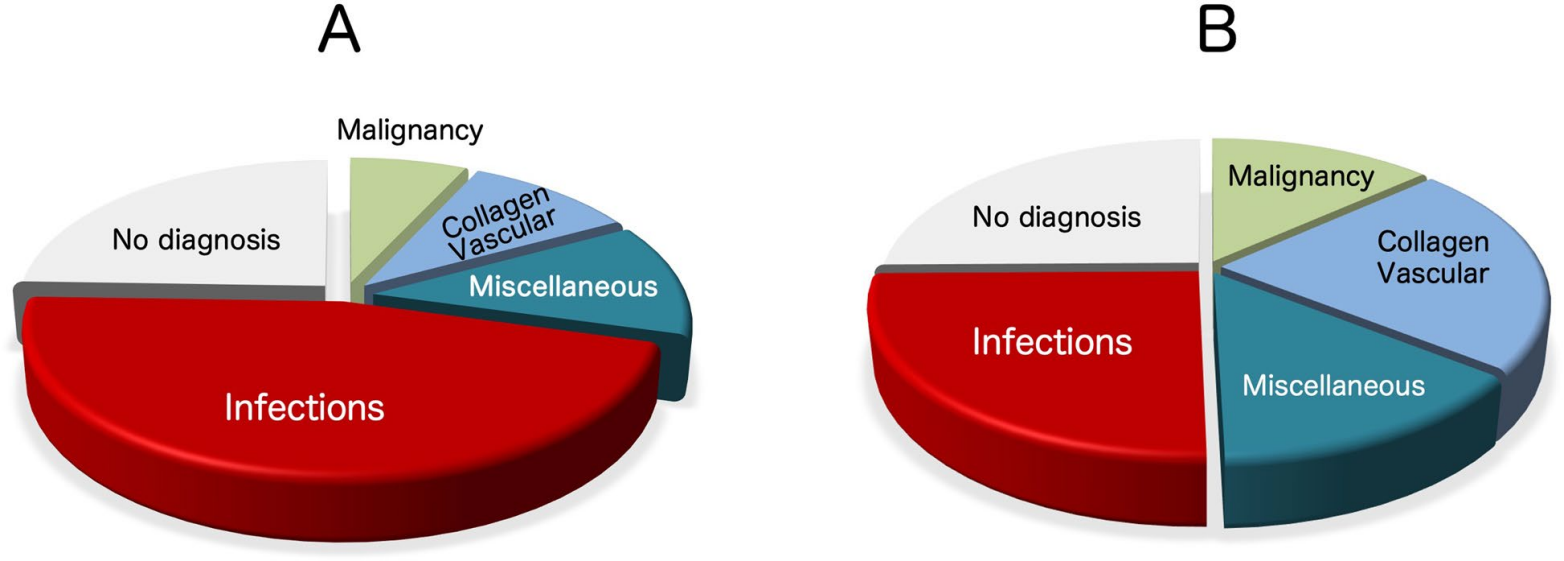
Final etiologies for fever of unknown origin. Collective data from multiple references are shown to compare the frequencies of final diagnoses for fever of unknown origin. Children (**A**) and adults (**B**) show different distributions

Several reports have analyzed the causes of recurrent or prolonged fever [1, 10–16]. Among children with febrile illnesses, infectious diseases comprised 46.2% of cases, whereas only 25.2% among adults involved infectious diseases. In contrast, the frequency of malignant illness was 6.9% among children and as high as 13.3% among adults (Fig. 1). Autoinflammatory diseases comprise only a minor proportion of febrile illnesses and are usually categorized as miscellaneous illnesses [17]. Due to the fact that acute infections are the most common cause of FUO among children, autoinflammatory diseases are frequently overlooked by pediatricians. The very low frequency of autoinflammatory diseases and the episodic (or transient) nature of the inflammatory symptoms further contribute to the difficulties of diagnosing pediatric autoinflammatory diseases. Regardless of the nature of the treatment, febrile symptoms will resolve within several days and pediatricians and then typically stop thinking about the true etiology of the inflammation unless symptoms recur frequently or are prolonged for an extended period.

### Autoinflammatory diseases

“Autoinflammatory disease” was proposed by McDermott et al. more than 20 years ago, when they described a monogenic periodic fever syndrome, tumor necrosis factor receptor-associated periodic fever syndrome (TRAPS) [2]. Since then, increasing numbers of diseases have been added to the category of autoinflammatory diseases [18, 19]. A substantial proportion of these diseases is caused by mutations in certain genes responsible for the regulation of inflammatory cascades. However, causative genes remain unidentified for many other inflammatory conditions.

The prototype autoinflammatory disease is FMF [20]. This pathology is most prevalent among east Mediterranean countries, including Armenia, Turkey, Greece, and Italy. In typical cases, FMF is characterized by a short episode of high fever, associated with pain in the chest, abdomen, or joints. In some cases, the patients may exhibit diffuse erythematous swelling of the ankle region of the legs. *MEFV* was identified as the gene most closely associated with FMF [21, 22]. Several disease-causing mutations within *MEFV* have been identified as responsible for the onset of typical cases of FMF. However, numerous other mutations were regarded as simple polymorphisms or, at most, disease-modifying missense mutations [23–25]. For this reason, the diagnosis of FMF still relies on the clinical profile, rather than mutation analyses, leaving much space for discussion to diagnose atypical cases of FMF [25, 26].

### FMF among children

Age at onset is highly variable for many autoinflammatory diseases. In some cases, disease onset occurs as early as infancy, whereas the first symptom may appear after 60 years old [27, 28]. To avoid unnecessary discomfort for patients going through a long period until the proper diagnosis is finally made, and to prevent possible complications such as renal failure due to amyloidosis, early diagnosis and start of appropriate therapeutic intervention are obviously crucial. In most cases of autoinflammatory disease, genetic analysis is a useful tool to reach the proper diagnosis. However, in many other cases, mutational analyses alone do not reliably allow correct diagnosis. This is particularly true for many of the polygenic autoinflammatory diseases and for a significant proportion of monogenic autoinflammatory diseases, such as FMF.

**Fig. 2:**
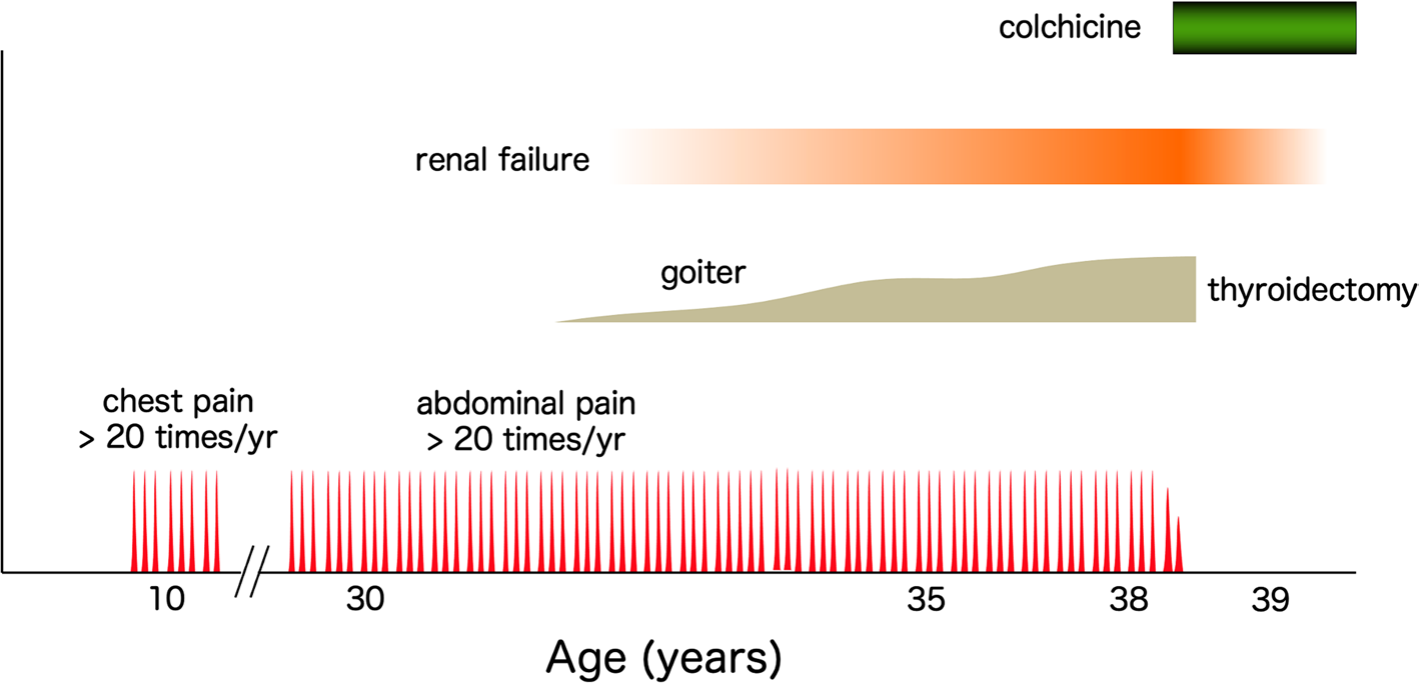
Diagnostic odyssey for a case with typical FMF. A representative case of typical FMF. The patient started to experience frequent episodes of high fever and chest pain at <10 years old. Similar attacks recurred numerous times over the following years. As he became older, chest pain was replaced by abdominal pain. The final diagnosis was made when renal dysfunction was identified at the time of thyroidectomy for goiter. Both renal dysfunction and goiter were later confirmed to be due to amyloidosis. After initiation of colchicine, the long-lasting episodes of fever and pain rapidly resolved

The onset of FMF is usually very early. In some cases, febrile episodes may start as early as only a few weeks after birth [29]. However, diagnosis is frequently delayed due to the absence of a typical clinical picture. Many reports have suggested that the younger the patient, the longer the diagnostic delay will be. Tamatar et al. recently reported the clinical features of FMF cases among children and showed that the average delay in diagnosis was 3.4 ± 3.2 years for children who experienced the first episode before 3 years old [30]. Diagnostic delay is much common in Asian countries where the prevalence of FMF is low. Migita et al. studied 126 cases of FMF in Japanese and concluded that the mean diagnostic delay was 9.1 ± 9.3 years [31]. Kishida et al. compared the diagnostic delay between different age groups in Japanese [32]. The mean diagnostic delays were 12, 4, and 3 years for age groups of ≤19 years old, 20–39 years old, and ≥40 years old, respectively. Sönmez et al. reported similar findings, with diagnostic delays of 7, 5.5, and 3 years for age groups of ≤5 years, 6–11 years, and ≥12 years, respectively [27]. Such results indicate that children suffer longer diagnostic delays than adults, regardless of the ethnic background. Furthermore, a prolonged diagnostic odyssey may be frequently experienced by children in Japanese, where the disease is not well known and the frequency is relatively low as compared to eastern Mediterranean countries. Figure 2 shows a representative case. The episodes of recurrent fever associated with abdominal pain started when the patient was younger than 10 years old. No diagnosis was made for his symptoms, and the febrile attacks continued for more than 30 years. FMF was finally diagnosed when he developed renal failure and swelling of the thyroid gland, both of which were due to amyloidosis. Soon after administration of colchicine was started, all symptoms subsided and the patient subsequently gained freedom from all symptoms.

In countries where FMF is not prevalent or well-known, reaching the correct diagnosis early in life is very difficult. The symptoms may be incomplete, a detailed family history is usually lacking, each episode may last only a few days, and all severe symptoms may disappear regardless of the nature of therapy. To avoid unnecessary discomfort and preventable complications of amyloidosis, the development of a multi-faceted approach to the diagnosis of rare inflammatory illnesses is important. In addition to detailed history-taking and physical examination, characterization of the inflammatory responses by cytokine profiling and extensive mutational analysis of candidate genes may offer powerful tools to hasten the diagnosis of childhood febrile illnesses.

### Childhood febrile illnesses and cytokine profiling

**Fig. 3:**
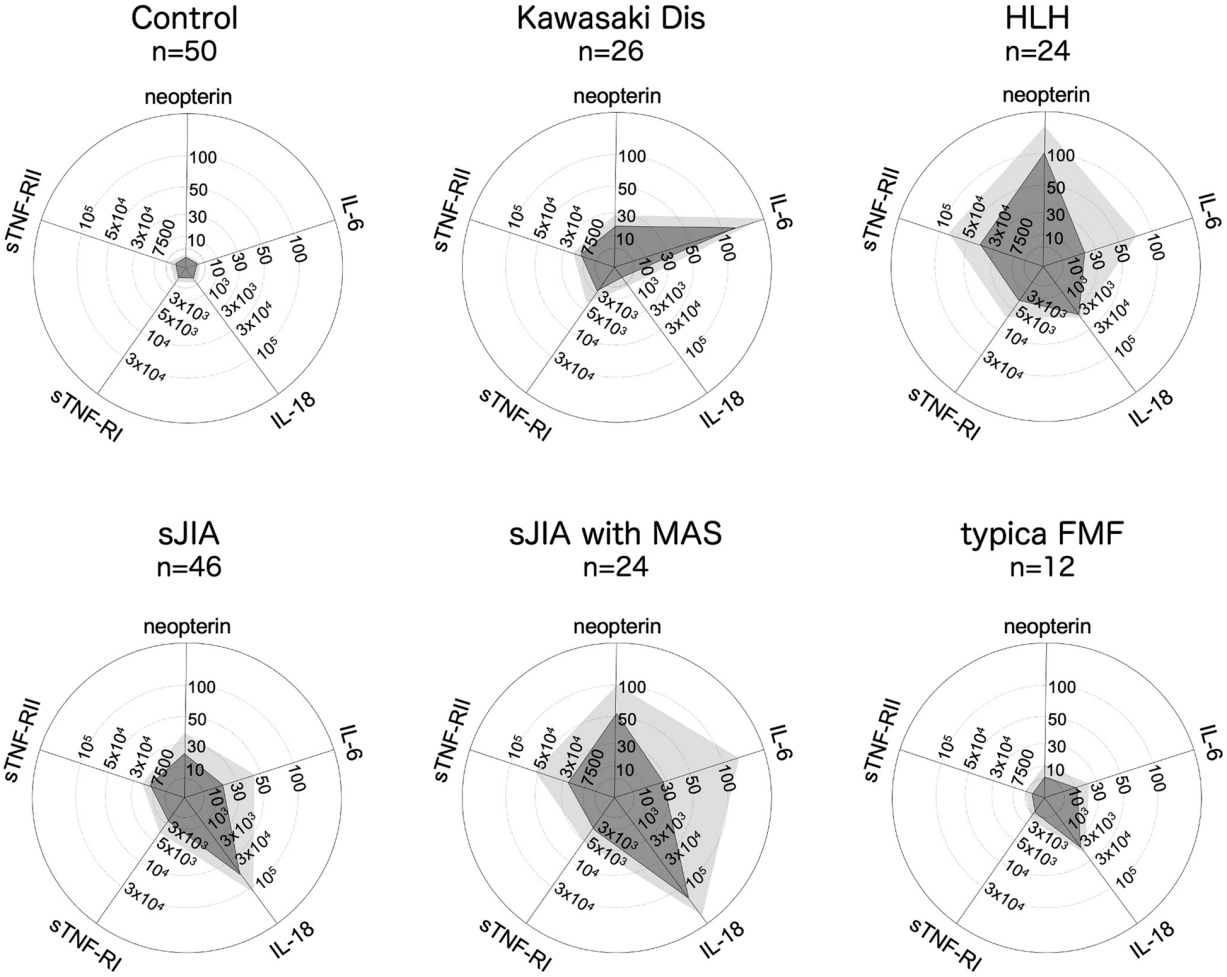
Cytokine profiles of inflammatory illness. Cytokine profiles of patients with different inflammatory illnesses. Neopterin, IL-6, IL-18, sTNF-RI, and sTNF-RII were measured at the same time, and the profiles are shown in radar charts. Dark gray pentagon shows the means, and light gray pentagon shows standard deviation + means of each data

Cytokine profiling, or simultaneous analysis of multiple inflammatory cytokines, offers objective information regarding the nature of the underlying disease process in many febrile illnesses. Various authors have reported characteristic cytokine profiles for different acute inflammatory illnesses [33–35]. The typical cytokine profiles of four different types of acute inflammatory illness are shown in Fig. 3. Typically, interleukin (IL)-6 is predominantly increased, whereas other inflammatory parameters remain within relatively low or normal levels in acute Kawasaki disease. In severe cases of Kawasaki disease, neopterin and IL-18 may also be increased. In contrast with Kawasaki disease, IL-18 is significantly elevated in the acute phase of sJIA, without exception, whereas IL-6 is increased at variable levels. The most important and potentially fatal complication of sJIA is macrophage activation syndrome (MAS), which is characterized by rapid progression of cytopenia, liver failure, dysregulation of the coagulation system, and finally, multi-organ failure. MAS is characterized by extreme elevations of inflammatory cytokines, with IL-6 as the most important. We reported previously that in addition to the high concentration of IL-6, the cytokine profile is characterized by further increases in IL-18, significant elevation of sTNF-RII in relation to sTNF-RI, and a high concentration of neopterin [36, 37]. In these reports, we showed that sTNF-RII-to-sTNF-RI ratio correlated positively with measures of the disease activity such as ferritin, AST, and LDH in patients with MAS associated with sJIA. In hemophagocytic lymphohistiocytosis (HLH), all inflammatory parameters are increased [34]. Among these parameters, increases in neopterin and an elevated sTNF-RII-to-sTNF-RI ratio are characteristic. In patients with typical FMF, serum IL-18 is increased during both febrile and afebrile periods, as described below.

### Pediatric autoinflammatory diseases associated with excessive IL-18 production

**Fig. 4:**
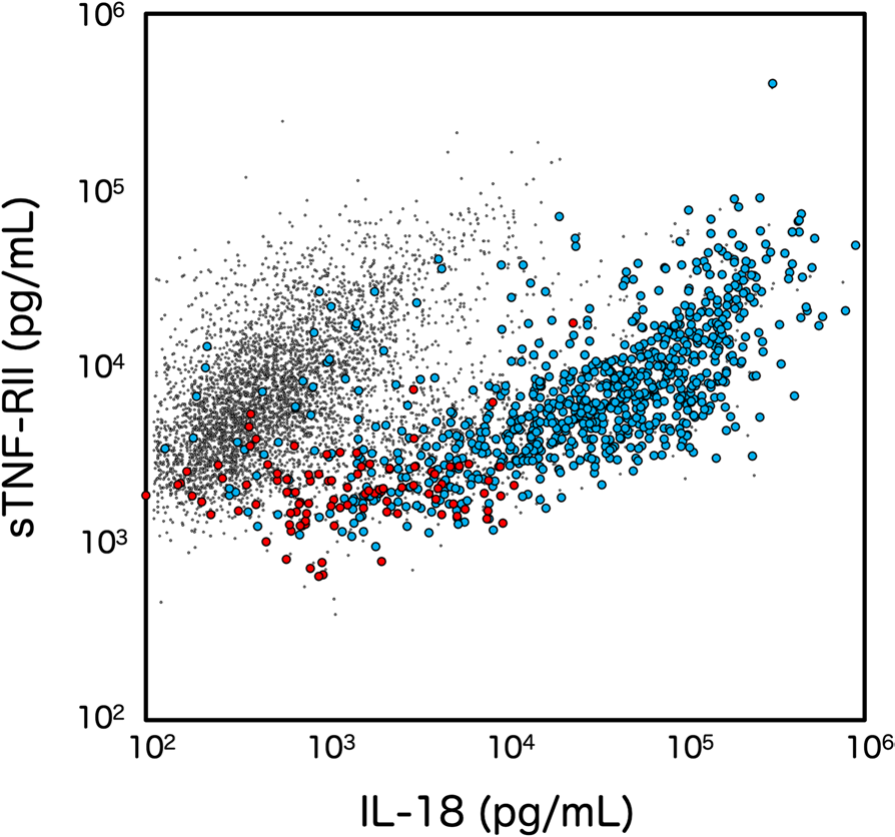
Plot analysis of sTNF-RII and IL-18 in cases with febrile illness. 9140 serum samples from febrile patients were examined for 5 different inflammatory parameters as described in the legend for Fig. 3. Clea Plot analyses of sTNF-RII and IL-18 are shown in the figure. Small gray dots show the samples from all febrile illnesses. Blue circles indicate patients with sJIA or AOSD. Red circles show the patients with typical FMF

Other than sJIA, serum IL-18 is markedly increased in some other diseases, most of which are associated with detrimental mutations to responsible genes. Among such diseases, X-linked inhibitor of apoptosis protein deficiency (XIAP) and activating NOD-like receptor family CARD-containing 4 protein mutation (NLRC4) are well-known immunodeficiency and autoinflammatory diseases [38, 39]. In both of these illnesses, the disease-causing genes are well-characterized and the illnesses are often associated with MAS, similar to sJIA. Patients with typical FMF show increased levels of IL-18, although the level is not as high as the abovementioned diseases [40, 41]. Figure 4 shows the relationship between sTNF-RII and IL-18 in 9140 patients with acute febrile illnesses. A positive correlation exists between sTNF-RII and IL-18 concentrations for most febrile patients. However, patients with sJIA and adult-onset Still’s disease (AOSD) show extremely elevated levels of IL-18. Although the levels of sTNF-RII increase in association with increased IL-18, the levels of IL-18 far exceed those of sTNF-RII in these patients. In patients with FMF, levels of IL-18 are also increased, but not as high as in sJIA or AOSD, and sTNF-RII usually remains within the normal range.

### Pathophysiology of FMF

**Fig. 5:**
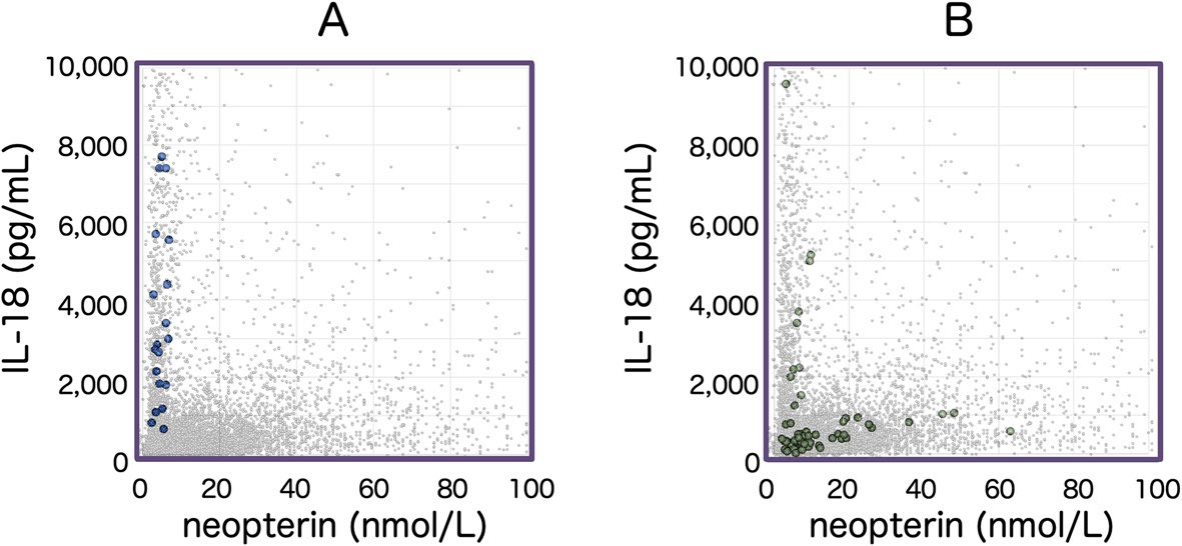
IL-18/neopterin plot patterns of typical and atypical FMF. Serum concentrations of IL-18 and neopterin were compared between 21 typical (**A**) and atypical 59 FMF (**B**) patients. Typical FMF patients consisted of patients with typical clinical manifestations and pathogenic mutations within exon 10 of *MEFV*. Atypical FMF patients had variable symptoms, and most of them had *MEFV* mutations within exon 2 and/or exon 3. Small dots represent data from 9060 control samples

In typical cases of FMF, periodic activation of the NLRP3 inflammasome is generally accepted to play a key role in the pathogenesis of the febrile episodes [42–44]. NLRP3 inflammasome activate caspase-1, which in turn cleave pro-IL-1beta and pro-Il-18 to mature form. As a result, serum IL-1β and IL-18 are elevated in these patients [45]. Elevated levels of IL-18 may serve as a crucial diagnostic clue together with the characteristic pattern of febrile episodes. In virtually all patients with typical FMF showing pathogenic mutations within exon 10 of the *MEFV* gene, serum IL-18 is elevated. Unless associated with severe amyloidosis, serum neopterin levels remain low or within the normal range (Fig. 5A). Although the levels of IL-18 are variable, the profiles are uniform and IL-18 is increased even during afebrile periods. Such data indicate that patients with typical FMF show a relatively characteristic inflammation. In contrast, patients with atypical FMF, with *MEFV* gene mutations within exon 2 and/or exon 3, show variable levels of IL-18 and neopterin, indicating that these patients comprise a heterogeneous disease population (Fig. 5B). Selective increases in serum IL-18 may provide diagnostic support for typical cases of FMF.

### Pathophysiology of sJIA

Although sJIA has been categorized under the diagnosis of JIA, distinct types of JIA with several different pathophysiologies exist [46]. Polyarticular or oligoarticular JIA is most likely compatible with adult-type rheumatoid arthritis (RA) [47]. sJIA is characterized by the significant increase of inflammatory cytokines, including IL-6, IL-1β, and IL-18 [48]. Most patients with sJIA have no family history and are the only individuals with this pathology among their relatives. For these reasons, sJIA is now regarded as an autoinflammatory disease with polygenic etiology [49]. AOSD shows similar clinical characteristics and cytokine profiles and may be regarded as closely related to sJIA [50, 51]. Both sJIA and AOSD comprise two subtypes with distinct clinical and laboratory characteristics [52, 53]. Arthritis subtypes include patients with a tendency to develop multi-joint inflammation, but with few episodes of macrophage activation. In contrast, systemic subtypes include patients with much less joint involvement, but a high frequency of MAS. IL-6, IL-17, and TNF-α is now generally accepted to play important roles in the pathogenesis of the arthritis subtype, whereas IL-1β, IL-18, and IFN-γ play central roles in the systemic subtype [54]. Understanding the roles of different cytokines in both subtypes suggests that different biologics may play important therapeutic roles in each type of sJIA or AOSD. Association of IL-1β/IL-18 with systemic subtype of sJIA/AOSD further supports the view that these cytokines play central role in the pathogenesis of macrophage activation in these diseases.

**Fig. 6:**
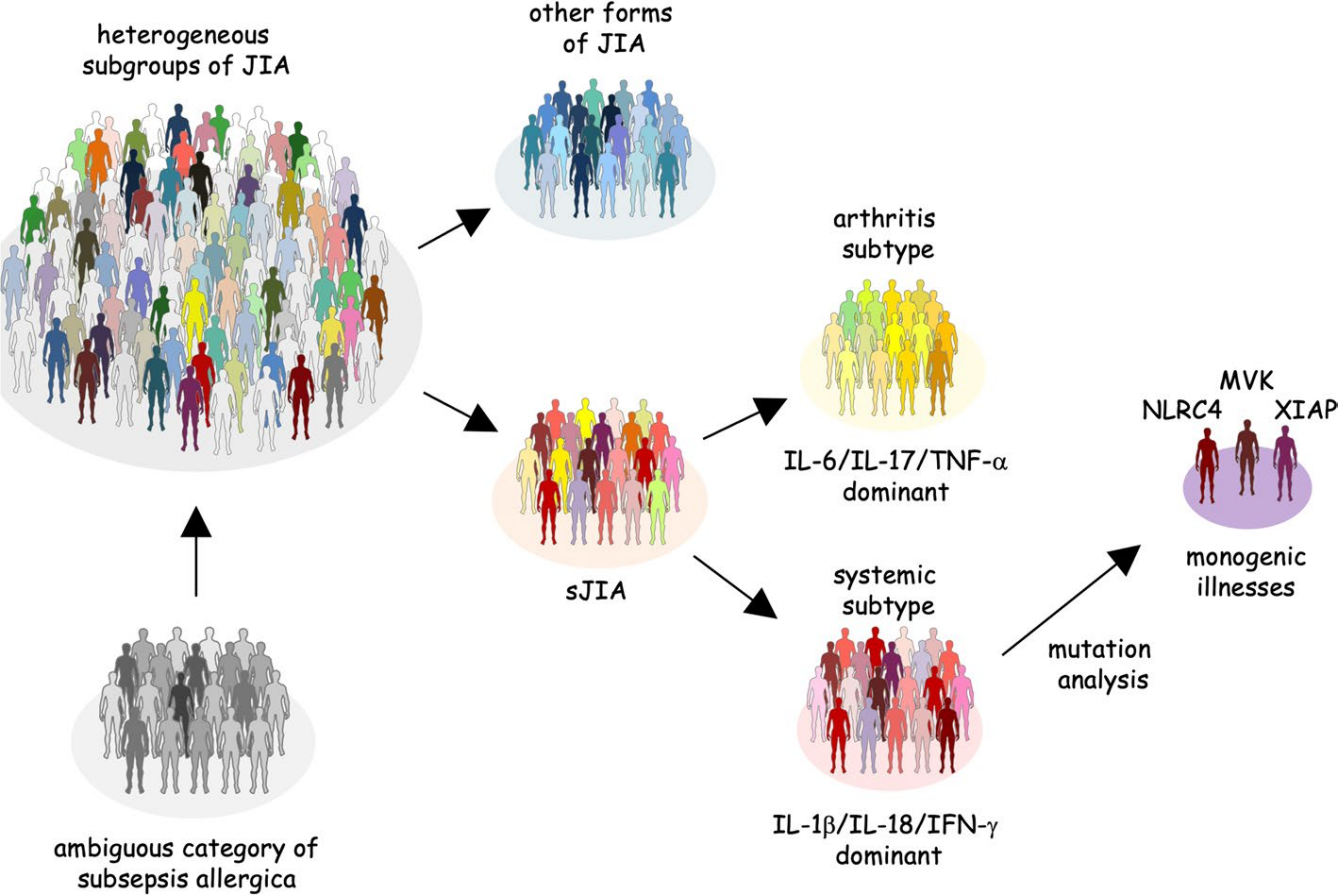
Subgroups of JIA and subtypes of sJIA. Schematic presentation of subgroups of JIA and subtypes of sJIA. Previously, many cases of fever of unknown origin with systemic manifestation were diagnosed as “subsepsis allergica,” when sJIA was yet to be used to describe such conditions

In rare cases of sJIA, particularly in systemic subtypes of sJIA, mutation analysis of certain genes may reveal pathogenic mutations. Among the genes responsible for clinical features similar to sJIA, NLRC4 and XIAP are known to show significantly elevated serum IL-18 and are frequently associated with MAS (Fig. 6) [38, 39, 55]. For these reasons, determining whether these genes are involved in the pathogenesis is of central importance whenever infants with sJIA or cases with high serum titers of IL-18 are encountered. By exploring the molecular pathogenesis of NLRC4 and XIAP, we may be able to understand the characteristic pathology of sJIA.

### IL-18 as a key modulator of inflammation and immune dysfunction

The precise mechanisms by which extremely elevated serum IL-18 leads to the characteristic clinical features observed in patients with sJIA remain unclear. However, recent publications have suggested that sustained elevation of serum IL-18 may be closely related to dysfunctions in natural killer (NK) cells [56]. Exposure of NK cells to high serum IL-18 results in activation and subsequent cell death of NK cells, leading to significantly decreased numbers of circulating NK cells [56, 57]. At the same time, intense activation of NK cells with an extremely high concentration of IL-18 may result in transient hyperproduction of interferon (IFN)-γ [58]. In accordance with these assumptions, patients with sJIA show impaired or absent NK activity during the acute phase of the illness [59]. In vivo exposure of NK cells to high concentrations of IL-18 results in significantly reduced response to exogenous IL-18, as circulating NK cells from acute-phase sJIA patients universally show absent activation response to in vitro stimulation [60]. Low or absent NK activity is the central feature of primary HLH (pHLH) [61, 62], which is characterized by genetic defects in the components of the cytotoxic machinery in NK cells and cytotoxic T cells. Due to the lack of NK activity, patients with pHLH are prone to excessive inflammatory cytokine production and progressive cytopenia. MAS, which is typically observed during the acute phase of sJIA, shows similar pathology, with the following points [63]:

1. Low or absent NK activity
2. Excessive cytokine production, particularly for IFN-γ
3. Progressive cytokinemia
4. Multi-organ dysfunction

Due to the rapidly fatal clinical course of MAS associated with sJIA, preventing its onset, reaching an early diagnosis and intervening as early as possible once the diagnosis is brought to mind are of paramount importance.

### FMF, sJIA, and related diseases with high serum IL-18

**Fig. 7:**
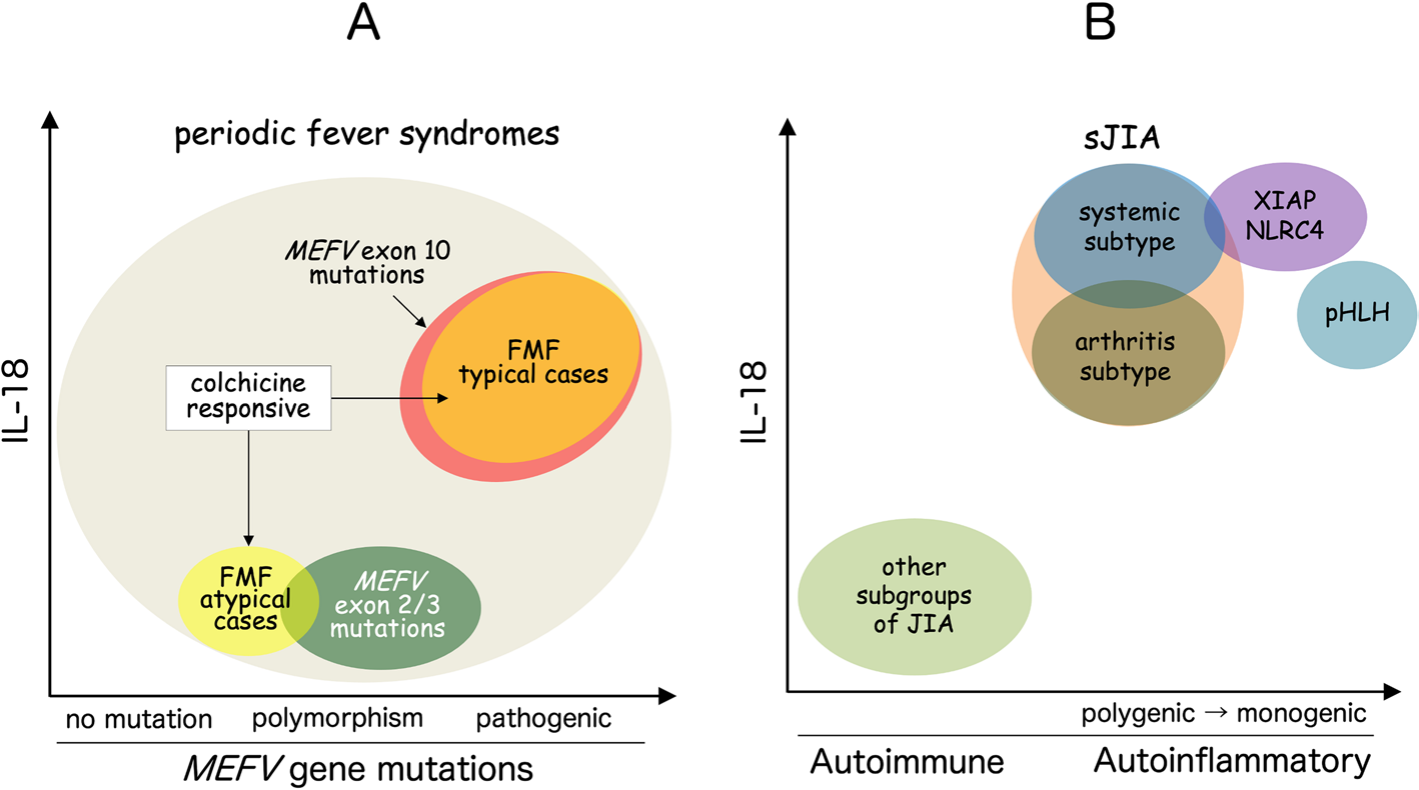
Schematic view of FMF and diseases with high serum IL-18. Schematic views of FMF and periodic fever syndromes (**A**) and sJIA/AOSD and related diseases (**B**) are shown. Typical cases of FMF (orange) consist of cases with characteristic clinical presentation, colchicine response, high serum IL-18, and pathogenic mutations within exon 10 of *MEFV* (red). Some cases of periodic fever syndromes may also respond to colchicine without exon 10 mutation of *MEFV* (yellow). These cases, with (dark green) or without mutations (ivory) of *MEFV*, are classified as atypical FMF. Other than FMF, sJIA, AOSD (blue), and several monogenic diseases, such as XIAP, NLRC4 (violet), and pHLH (navy) are characterized by extremely elevated serum IL-18 and they are prone to develop MAS

Among patients with periodic fever syndromes, FMF can be diagnosed from different perspectives, including clinical characteristics, *MEFV* mutation analysis, and cytokine profiles. A multi-faceted approach will facilitate precise diagnosis early in life. The relationships between these perspectives and different diseases are shown in Fig. 7A. High serum IL-18 levels reflect sustained activation of the NLRP3 inflammasome, a characteristic of typical cases of FMF. Although patients with typical FMF show high serum IL-18 without exception, atypical cases usually show normal levels of IL-18. A small fraction of FMF with exon 10 mutations may not respond to colchicine [64]. Atypical cases of FMF may also respond to colchicine, but *MEFV* mutation analyses fail to show mutations in exon 10.

A schematic view of sJIA and related illnesses is shown in Fig. 7B. Extremely elevated levels of serum IL-18 are a characteristic feature of sJIA or AOSD, as polygenic autoinflammatory diseases. High serum IL-18, together with other clinical characteristics, distinguishes sJIA from other subgroups of JIA. In addition, sJIA is further divided into two subtypes. The systemic subtype shows higher IL-18 levels and is more frequently complicated by MAS than the arthritis subtype. Significantly increased IL-18 levels are also seen in the monogenic immunological diseases, including XIAP, NLRC4, and pHLH. Regardless of the causes, high IL-18 represents an alarming sign of an impending risk of macrophage activation and progressive development of multi-organ failure.

## Conclusions

Whenever children with prolonged or recurrent febrile episodes are encountered, a detailed clinical history must be elicited and a thorough clinical examination performed. Determination of serum cytokines offers a significant clue to the understanding of the nature of inflammatory illnesses. IL-18, in particular, is useful for the diagnosis of sJIA and FMF. The serum levels of IL-18 may indicate the risk of MAS in sJIA. Furthermore, the finding of elevated IL-18 levels in infants may lead to a genetic diagnosis of XIAP or NLRC4. A multi-faceted approach to the diagnosis of patients with FUO is necessary for both patients and their families to avoid an unnecessary diagnostic odyssey.

## Data Availability

Any materials in support of this manuscript are available on reasonable request.

## Abbreviations

FUO: Fever of unknown origin
FMF: Familial Mediterranean fever
sJIA: Systemic juvenile idiopathic arthritis
TRAPS: Tumor necrosis factor receptor-associated periodic fever syndrome
IL: Interleukin
MAS: Macrophage activation syndrome
AOSD: Adult-onset Still’s disease
IFN-γ: Interferon-γ
pHLH: Primary hemophagocytic lymphohistiocytosis
NLRP3: Nucleotide-binding oligomerization domain-like receptor family, pyrin domain-containing 3
NLRC4: NOD-like receptor family CARD-containing 4 protein
XIAP: X-linked inhibitor of apoptosis protein
